# COVID-19 point-of-care tests can identify low-antibody individuals: In-depth immunoanalysis of boosting benefits in a healthy cohort

**DOI:** 10.1126/sciadv.adi1379

**Published:** 2024-06-12

**Authors:** Michael Mallory, Jennifer E. Munt, Tara M. Narowski, Izabella Castillo, Edwing Cuadra, Nora Pisanic, Paul Fields, John M. Powers, Alexandria Dickson, Rohan Harris, Richard Wargowsky, Seamus Moran, Ahmed Allabban, Kristin Raphel, Timothy A. McCaffrey, James D. Brien, Christopher D. Heaney, John E. Lafleur, Ralph S. Baric, Lakshmanane Premkumar

**Affiliations:** ^1^ Department of Microbiology and Immunology, University of North Carolina School of Medicine, Chapel Hill, NC, USA.; ^2^ Department of Epidemiology, UNC Chapel Hill School of Public Health, University of North Carolina at Chapel Hill, Chapel Hill, NC, USA.; ^3^ Department of Environmental Health and Engineering, Johns Hopkins Bloomberg School of Public Health, Baltimore, MD, USA.; ^4^ Adaptive Biotechnologies, Seattle, WA, USA.; ^5^ Department of Molecular Microbiology and Immunology, Saint Louis University, Saint Louis, MO, USA.; ^6^ Department Emergency Medicine, George Washington University School of Medicine, Washington, DC, USA.; ^7^ Department of Medicine, Division of Genomic Medicine, The George Washington University Medical Center, Washington, DC, USA.; ^8^ Department of International Health, Bloomberg School of Public Health, Johns Hopkins University, Baltimore, MD, USA.

## Abstract

The recommended COVID-19 booster vaccine uptake is low. At-home lateral flow assay (LFA) antigen tests are widely accepted for detecting infection during the pandemic. Here, we present the feasibility and potential benefits of using LFA-based antibody tests as a means for individuals to detect inadequate immunity and make informed decisions about COVID-19 booster immunization. In a health care provider cohort, we investigated the changes in the breadth and depth of humoral and T cell immune responses following mRNA vaccination and boosting in LFA-positive and LFA-negative antibody groups. We show that negative LFA antibody tests closely reflect the lack of functional humoral immunity observed in a battery of sophisticated immune assays, while positive results do not necessarily reflect adequate immunity. After booster vaccination, both groups gain depth and breadth of systemic antibodies against evolving SARS-CoV-2 and related viruses. Our findings show that LFA-based antibody tests can alert individuals about inadequate immunity against COVID-19, thereby increasing booster shots and promoting herd immunity.

## Teaser

Point-of-care antibody tests can detect inadequate immunity.

## INTRODUCTION

By the end of 2022, more than 96% of US individuals aged 16 or older had severe acute respiratory syndrome coronavirus 2 (SARS-CoV-2) antibodies from infection or vaccination (*1*, *2*). However, immunity against SARS-CoV-2 tends to wane over time (*3*) and is typically less effective against evolving SARS-CoV-2 variants (*4*). Individuals with weaker neutralizing responses against the Wuhan strain showed reduced protection with SARS-CoV-2 variants (*5*). Major SARS-CoV-2 variants, such as Delta and Omicron, have accumulated mutations within the receptor binding domain (RBD) of the spike protein. These mutations alter spike protein antigenic properties and affect vaccine-induced antibody (Ab) binding and neutralizing activities (*6*). Some spike RBD mutations enhanced viral interaction with the host cell surface receptor, angiotensin-converting enzyme 2 (ACE2) (*7*).

As the Omicron subvariants emerged, the information about the waning immunity following vaccination caused a major concern (*8*, *9*). Yet, millions of eligible individuals have not opted to receive the third booster dose targeting ancestral Wuhan spike protein (*10*). Although widely available, far fewer have received the newly approved booster vaccines targeting Omicron subvariants (*10*). Various factors may contribute to booster hesitancy (*11*–*13*), including questions about booster effectiveness against newer, emerging variants. Some individuals developed vaccine-induced side effects (*14*–*17*), which may also influence decisions about the risks/benefits of vaccinating. Thus, it is crucial to implement strategies that increase vaccine confidence and encourage vaccine uptake to protect individuals with waning or weakened immunity against evolving SARS-CoV-2 strains.

We previously reported on mRNA vaccine–induced Ab responses in a health care provider (HCP) cohort in which many healthy participants developed highly variable neutralizing Abs after two-dose vaccination (*4*). In the same HCP cohort, here, we report on performance of low-cost point-of-care (POC) tests in individuals with weak vaccine responses. We tested the ability of serum and oral fluid lateral flow POC tests to detect inadequate immunity as reflected in low SARS-CoV-2 spike Ab levels and found that negative POC test results strongly correlated with weak functional neutralizing Ab levels while positive Ab test is not evidence for protective immunity. We then investigated the breadth and depth of blood and salivary Abs and T cell clonal response among POC test–positive and POC test–negative individuals before and after the third booster dose and found significant increases in both groups–the POC Ab test–positive group had more Abs than the negative group. Our study suggests that the convenient, low-cost POC Ab tests are a simple tool for detecting individuals with low Ab levels. Those with inadequate immunity may use this information in decision-making around getting booster vaccinations.

## RESULTS

### SARS-CoV-2 Ab response decline >4 months after the second-dose of the mRNA vaccine

**Fig. 1.**
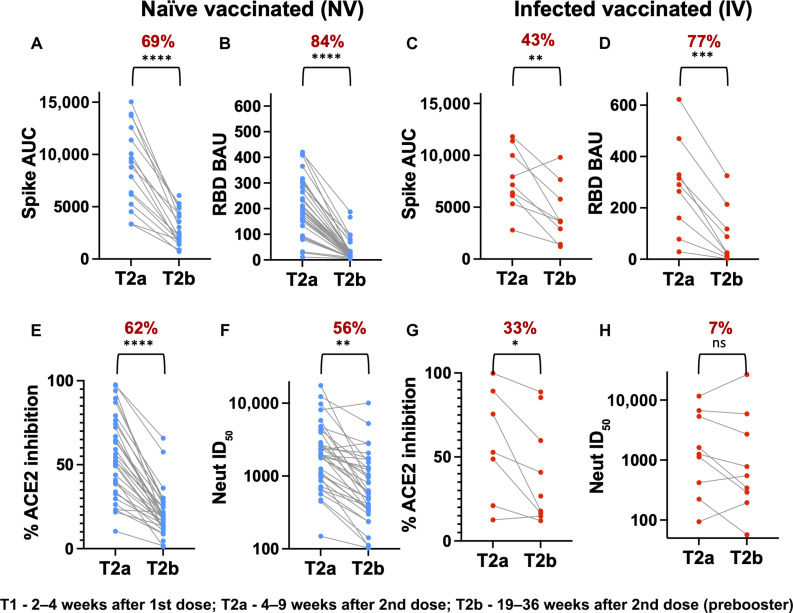
Dynamics of Ab responses to Wuhan strain after mRNA vaccine. Analysis of Spike (**A** and **C**) or RBD (**B** and **D**) binding Abs, ACE2 blocking Abs (**E** and **G**), and neutralizing Abs (**F** and **H**) to the Wuhan strain between 4 and 9 weeks (T2a) and 19 and 36 weeks (T2b) after dose 2. NV subjects (*n* = 35) and IV subjects (*n* = 9) are shown in blue and red dots, respectively. The mean percent decrease in Ab levels and paired *t* test summary {0.12 [not significant (ns)], 0.0332 [*], 0.0021 [**], 0.0002 [***], and <0.0001 [****]} between the two time points are shown in brackets above the plot. AUC, area under the curve; BAU, binding Ab unity; ID_50_, 50% inhibitory dose.

To evaluate the dynamics of binding and functional Abs after a two-dose mRNA vaccine based on Wuhan strain of SARS-CoV-2, we analyzed samples collected at two time points post-second dose: T2a (4 to 9 weeks) and T2b (19 to 36 weeks) from 35 naïve-vaccinated (NV) and 9 infected-vaccinated (IV) subjects in the HCP cohort. We measured spike and RBD binding, RBD-ACE2 blocking Ab activity, and infectious virus neutralization activity toward the Wuhan strain using the methods previously established (Fig. 1) (*4*, *18*). Spike and RBD binding Ab levels declined 43 to 77% between T2a and T2b in both the NV and IV subjects (Fig. 1, A to D). RBD-directed Abs decreased more than overall spike-directed Abs (*19*), regardless of NV or IV status. NV subjects also showed a comparable reduction in ACE2 blocking Abs (Fig. 1E) and SARS-CoV-2 neutralizing activities, 62 and 56%, respectively (Fig. 1F) (*4*). However, the decline among IV subjects was more modest for ACE2 blocking Ab (33% change, *P* = 0.0332) (Fig. 1G) and insignificant for neutralizing Abs (7% change; Fig. 1H) during this period. Thus, our data suggest that the mRNA vaccine–induced Ab response declines substantially >4 months following the second dose of the mRNA vaccine in our HCP cohort (*20*).

### Low-cost Rapid LFA blood test informs low circulating functional Abs

**Fig. 2.**
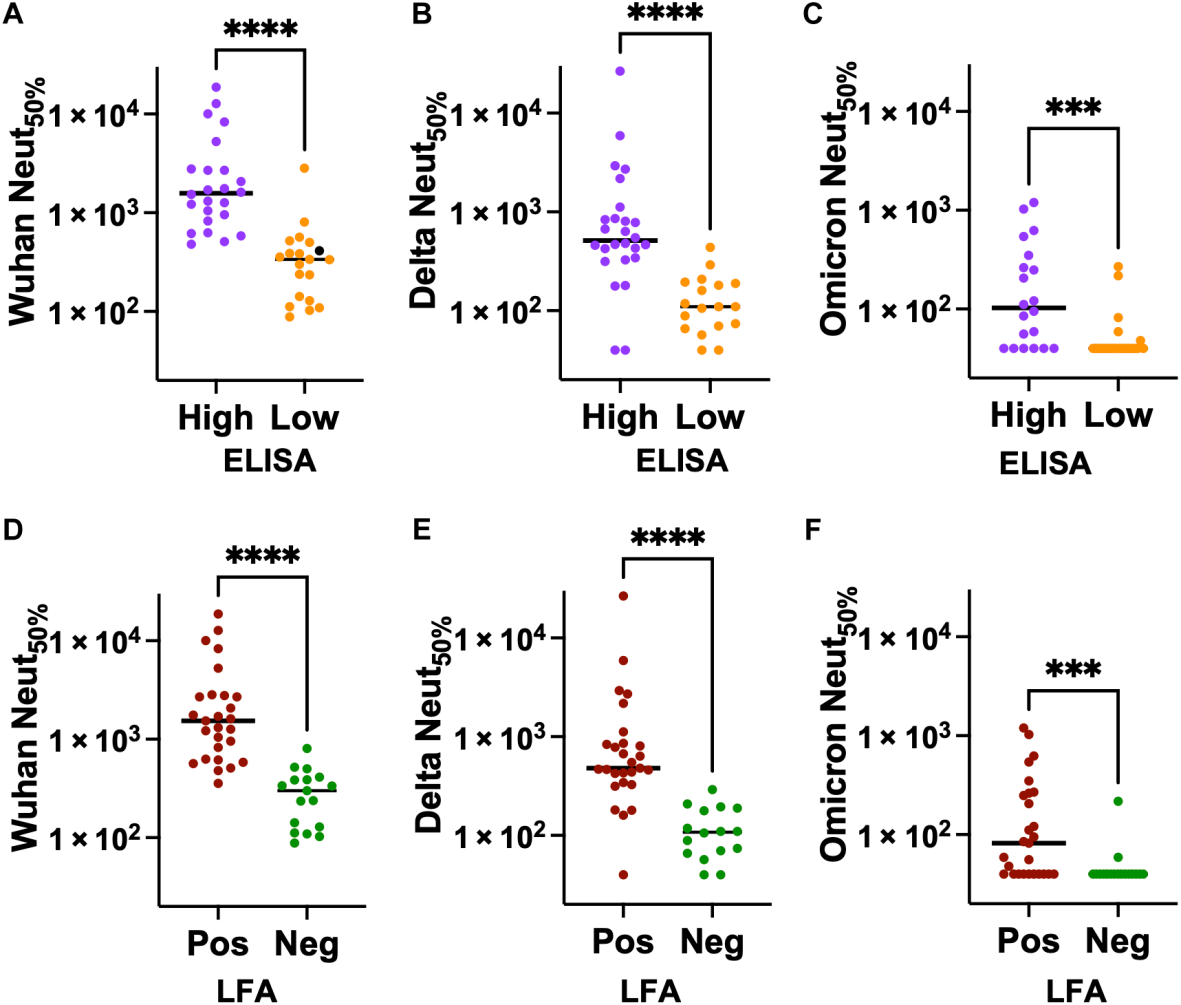
Low-cost Rapid LFA blood test to detect low circulating Abs. Assess low ELISA RBD binding agreements with the weak neutralization activity against (**A**) Wuhan, (**B**) Delta, and (**C**) Omicron strain. Assess negative LFA blood test agreements with neutralizing titers of (**D**) Wuhan, (**E**) Delta, and (**F**) Omicron strains. The *P* value summary from the Mann-Whitney nonparametric unpaired test to determine the difference between the groups is shown above the scatterplot. ****P < 0.0001; ***P = 0.0008.

To help build tools to support informed decision-making about the COVID-19 booster vaccination, we conducted experiments to investigate whether simple laboratory binding assays (*21*, *22*) could inform the lack of functional neutralizing Abs (*23*, *24*) in blood samples. Our initial evaluation involved determining whether our RBD enzyme-linked immunosorbent assay (ELISA) binding results stratified by low and high could predict the weak infectious live-virus neutralizing activity (Fig. 2, A to C) in blood samples collected at T2b (19 to 36 weeks after dose 2). As has been previously reported (*25*–*27*), the lowest RBD binders aligned well with weak neutralizing, as indicated by the high statistical significance (*P* < 0.0001).

Next, we tested whether a low-cost lateral flow POC test could predict the weak live-virus neutralization in blood samples. As a proof of concept, we evaluated the emergency use authorized (EUA) Cellex qSARS-CoV-2 immunoglobulin G (IgG)/IgM Rapid test, which uses the RBD of the Wuhan strain and can be performed with a drop of blood (~10 μl) which can be read in less than 20 min (fig. S1). As described before (*28*), our initial assessment of the negative test result of the Cellex Rapid test highly correlated with low RBD binding activity (*P* < 0.0001), which warranted further evaluation with functional assays. Next, we assessed whether the Rapid test results, separated into negatives and positives, could inform weak neutralizing activities against the Wuhan, Delta, and Omicron strains using blood samples collected at T2b from 44 subjects (Fig. 2). For both Wuhan and Delta strains, the negative Rapid test results were highly correlated with the weak live-virus neutralization activity with high statistical significance (*P* < 0.0001) (*29*). However, when it comes to the more divergent Omicron strain, the correlation between the Rapid test results and the live-virus neutralizing activity was moderately low (*P* = 0.008), indicating the need for using LFAs that use the Omicron antigen to improve accuracy (*30*). Next, we aimed to assess the accuracy of the LFA test in determining the 50% protective neutralization titer, which is the level of neutralization required for an individual to have 50% protective efficacy. We used the Wuhan neutralization titer 512 as a threshold, representing 50% of the reported correlate of protection titer for mRNA vaccine against SARS-CoV-2 variant infection (*31*). Accordingly, we found that the LFA test had a positive agreement of 92.31% [confidence interval (CI): 75.86 to 98.63%] and a negative agreement of 83.33% (CI: 60.78 to 94.16%) compared to the live-virus neutralization assay.

### Low-cost Rapid LFA oral fluid informs low circulating functional Abs

**Fig. 3.**
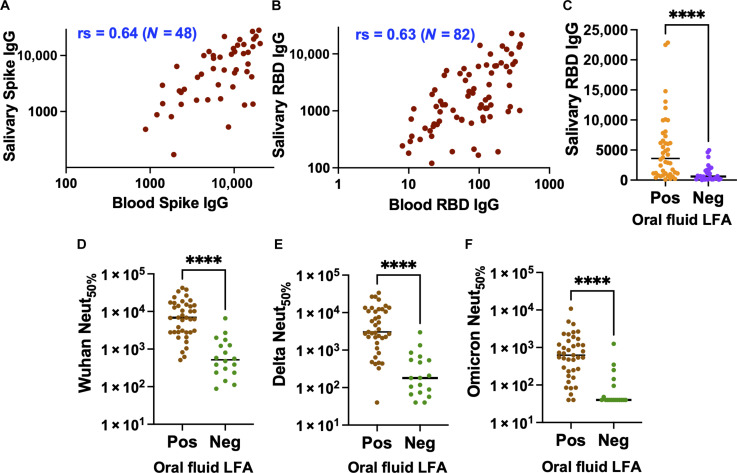
Low-cost Rapid LFA oral fluid test to detect low circulating Abs. Assess the correlation between blood and oral fluid IgG against (**A**) Spike and (**B**) RBD. The nonparametric Spearman correlation coefficients (rs) for the plots are shown. (**C**) Assess low Luminex RBD binding agreements with the negative LFA oral fluid test. Assess negative LFA oral fluid test agreements with neutralizing titers of (**D**) Wuhan, (**E**) Delta, and (**F**) Omicron strains. The *P* value summary from the Mann-Whitney nonparametric unpaired test to determine the difference between the groups is shown above the scatterplot. ****P < 0.0001.

As oral fluid samples are an attractive alternative to blood testing due to their noninvasive nature and ready repeatability, we assessed the correlation between the Wuhan spike and RBD binding Abs in blood and oral fluid samples (Fig. 3, A and B). Both spike and RBD IgG from oral fluid and blood were strongly correlated despite being measured by two independent methods (Luminex bead assay versus ELISA), as oral fluid IgG primarily derives from plasma by transudation from the gingival blood circulation (*32*–*34*). As a proof of concept, we assessed the EUA CovAb SARS-CoV-2 IgG test, which measures RBD IgG in 15 min using oral fluid (gingival crevicular fluid; fig. S2). The correlation between the negative test results by the CovAb IgG test and the low RBD binding activity measured with blood aligned (*35*) with high significance (*P* < 0.0001) (Fig. 3C). The negative CovAb test results were highly correlated with the weak live-virus neutralizing activities for the Wuhan, Delta, and Omicron strains with high significance (*P* < 0.0001) (Fig. 3, D to F). Applying the cutoff value of 50% of the correlate of protection titer (512) described above, the oral fluid-based LFA test showed a positive agreement of 80.85% (CI: 67.46 to 89.58%) and a negative agreement of 90.00% (CI: 59.58 to 99.49%). The wide CIs in the negative agreement result from the limited availability of the oral fluid samples from our cohort. These results support that the LFA platforms using oral fluid or blood samples can inform individuals of weak SARS-CoV-2 functional, especially neutralizing, Ab responses.

### Booster enhances SARS-Co-2 IgG response in both LFA-positive and LFA-negative groups

**Fig. 4.**
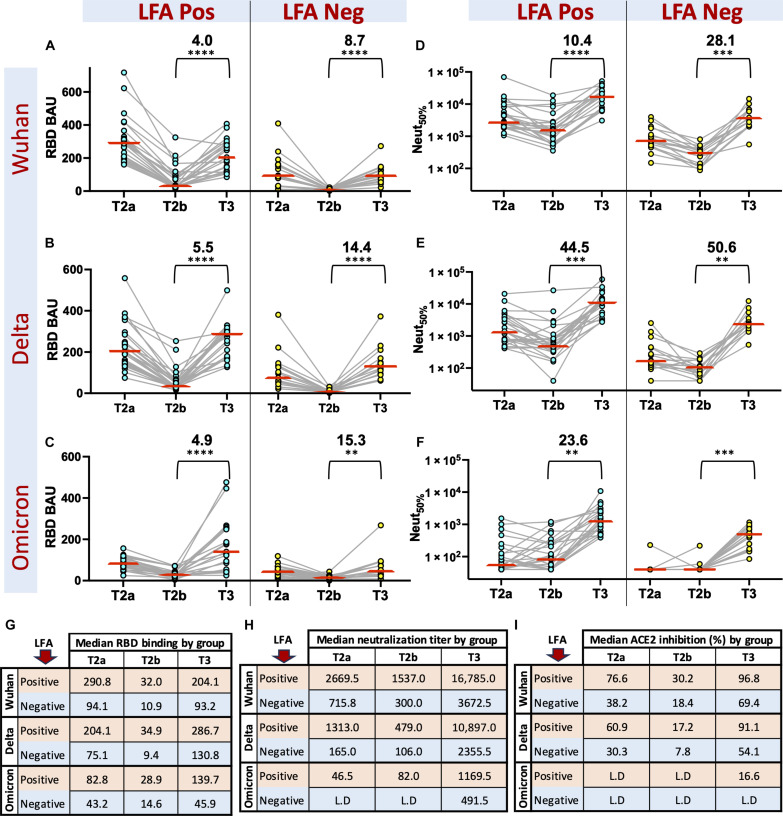
Dynamics of Ab responses in LFA-negative and LFA-positive groups before and after the third booster dose. Analysis of (**A** to **C**) RBD binding and (**D** to **F**) neutralizing Abs to the Wuhan, Delta, and Omicron variants in LFA-positive and LFA-negative individuals at T2a (4 to 9 weeks after dose 2), T2b (19 to 36 weeks after dose 2), and T3 (3 to 18 weeks after dose 3). Median RBD binding Abs (**G**), neutralizing Ab titers (**H**), and ACE2 inhibiting Abs (%) (**I**) to the Wuhan, Delta, and Omicron variants in LFA positive (red rows) and LFA negative (blue rows) at T2a, T2b, and T3 time points. LFA-positive (*n* = 20) and LFA-negative (*n* = 13) groups were classified on the basis of the LFA results of the blood samples collected at T2b. The mean fold change in SARS-CoV-2 Abs and paired *t* test summary 0.0021 (**), 0.0002 (***), and <0.0001 (****) before (T2b) and after the booster (T3) in the LFA-positive and LFA-negative groups are shown in brackets above the plot. L.D, Limit of detection.

Next, we evaluated the vaccine-induced Abs in the LFA-positive and LFA-negative groups by tracking the dynamics of Spike RBD binding, live-virus neutralization activity, and ACE2 inhibition activity against the Wuhan, Delta, and Omicron variants at three time points: T2a, T2b, and T3 (Fig. 4, A to F, and fig. S3). After mRNA vaccination, both LFA-positive and LFA-negative groups showed a decline in levels of Ab binding, ACE2 inhibition activity, and neutralizing titers between T2a and T2b (Fig. 4, G to I). Likewise, the booster dose stimulated binding and neutralizing Abs in both groups albeit we observed no evidence for induction of salivary secretory IgA (sIgA) (fig. S4). The LFA-positive group had 1.9- to 3.7-fold higher binding antibodies and 2.4- to 8.0-fold higher neutralizing antibodies than the LFA negatives at every time point, but the fold Ab raise from T2b to T3 was distinctly higher for LFA negatives than the LFA positives (Fig. 4). Furthermore, the LFA-negative group had no detectable levels of neutralizing Abs or salivary antibodies targeting RBD (Fig. 4 and fig. S3) for the highly varying Omicron strain before receiving the booster. Thus, our data suggest that the LFA-negative group must receive booster vaccinations to develop neutralizing antibodies against the Omicron strain. However, both the LFA-positive and LFA-negative groups can benefit from the booster vaccine.

### Third booster dose enhances the avidity and breadth of the Abs

**Fig. 5.**
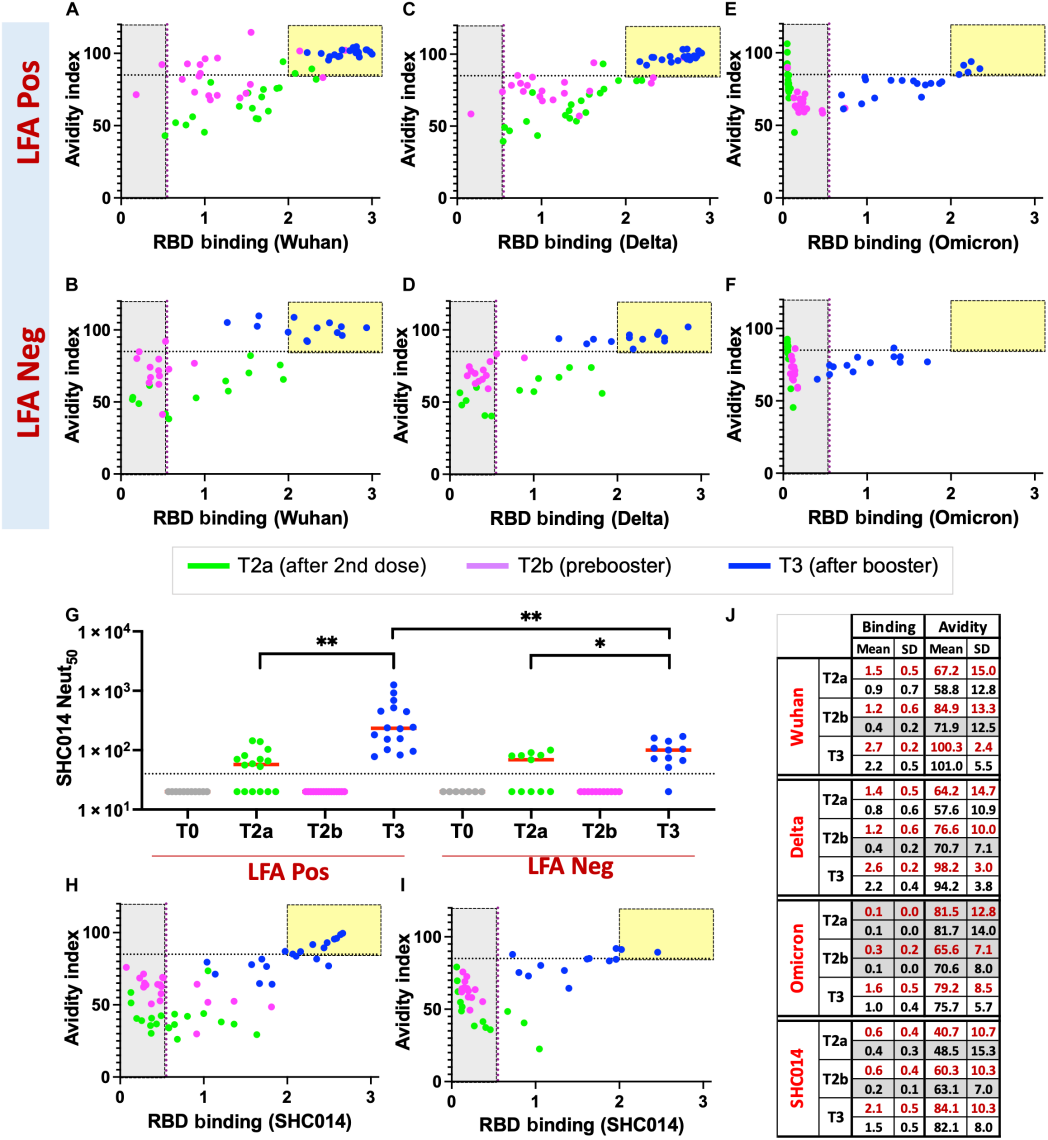
Avidity and breadth of the SARS-CoV-2 Abs. Assessment of Ab binding strength following mRNA vaccine against RBD from (**A** and **B**) Wuhan, (**C** and **D**) Delta, (**E** and **F**) Omicron, and (**H** and **I**) sarbecovirus bat SHC014 strains in LFA-positive and LFA-negative individuals at different time points. The avidity index, calculated by measuring Ab binding after treatment with urea solution relative to without urea treatment, has been plotted against binding without urea treatment in LFA-positive (*n* = 20) and LFA-negative (*n* = 13) groups. The yellow box indicates elevated levels of high-avidity Abs in plasma. Samples analyzed at respective prevaccine and postvaccine time points [T2a, 4 to 9 weeks after dose 2 (green); T2b, 19 to 36 weeks after dose 2 (pink); and T3, 3 to 18 weeks after dose 3 (blue)] are shown. (**G**) Analysis of neutralizing Ab levels against sarbecovirus bat SHC014 strain at different time points as above in LFA-positive (*n* = 16) and LFA-negative (*n* = 11) subjects. Paired *t* test summary between T2a and T3 [0.0027 (**) and 0.0125 (*)] and the Mann-Whitney test summary between LFA-positive and LFA-negative groups at T3 [0.0018 (**)] are shown in brackets above the plot. (**J**) RBD binding and avidity index summary for the four strains at three sampling points (T2a, T2b, and T3). The mean and SDs for LFA-positive (red text) and LFA-negative (black text) subjects are summarized. The low RBD binding activity renders the avidity index indeterminate (shown in gray).

Next, to assess the overall strength of RBD-directed antibodies developed after the booster (*36*) in LFA-positive and LFA-negative groups, we measured avidity (“functional affinity”) before (T2a and T2b) and after the third booster dose (T3) against the RBD of the Wuhan, Delta, and Omicron strains (*37*). To do this, we used a modified RBD ELISA, which uses 4 M urea as a chaotropic agent to dissociate low-avidity Abs from RBD while allowing only high-avidity Abs to remain associated. The ratio of the Abs bound with and without urea was used to express the avidity index (Fig. 5). In the LFA-positive group, the avidity index of RBD antibodies showed improvement for both Wuhan and Delta strains from T2a to T2b, despite a moderate decrease in RBD Ab levels in plasma during this period (Fig. 5, A, C, and J). The avidity index of RBD Ab for both Wuhan and Delta strains was comparable between the LFA-negative and the LFA-positive groups. However, the levels of RBD antibodies were lower in the LFA-negative group at T2a and eventually decreased below detectable levels at T2b (Fig. 5, B, D, and J). Omicron binding was low before the third booster dose in both these groups, rendering the avidity index indeterminate (Fig. 5, E, F, and J). After the third booster dose, plasma Ab levels toward all strains were much higher than observed at T2a and T2b in both LFA-positive and LFA-negative groups. At the same time, the Wuhan and Delta RBD Ab avidity index further improved to levels notably higher than observed at the earlier time points. Notably, the Omicron RBD Ab avidity index and the Ab levels in plasma after the third booster dose reflected the levels observed for the Wuhan and Delta strains at T2a in both these groups (Fig. 5).

To gain insights into the breadth of mRNA vaccine–induced Abs before (T2a and T2b) and after the third booster dose (T3), we measured neutralizing Abs to a bat SARS-like coronavirus RsSHC014 (SHC014), a phylogenetically distant sarbecovirus that uses the ACE2 receptor. In both the LFA-positive and LFA-negative groups, we observed a low SHC014 neutralizing Ab titer after T2a, which then declined to an undetectable level at T2b (Fig. 5G). After the booster, the SHC014 neutralizing Ab titer was higher than at T2a, although the median SHC014 neutralizing Ab titer in the LFA-positive group was marginally higher than in the LFA-negative group (234 versus 100). Next, we evaluated the RBD Ab avidity index and binding for SHC014 at T2a, T2b, and T3 time points (Fig. 5, H and I). In agreement with the SHC014 neutralizing Ab titers, the low-avidity RBD Abs developed after the second dose of the mRNA vaccine waned by T2b. After the booster, the RBD Ab avidity index and the Ab levels in plasma for the SHC014 were marginally better than for Omicron in the LFA-positive and LFA-negative groups, respectively (Fig. 5J).

We have also observed the elevation of spike Abs to seasonal human coronaviruses (HCoVs) after the first dose of the SARS-CoV-2 mRNA vaccine in our cohort (fig. S5) (*4*). However, these cross-reactive binding Abs do not contribute to the cross-neutralization, as evidenced by the neutralization titers between baseline (T0) and after the first dose (T1) for NL63 (seasonal α-HCoV) and OC43 (seasonal β-HCoV) (fig. S5).

### Lack of discernible difference in cellular response among LFA-positive and LFA-negative groups

**Fig. 6.**
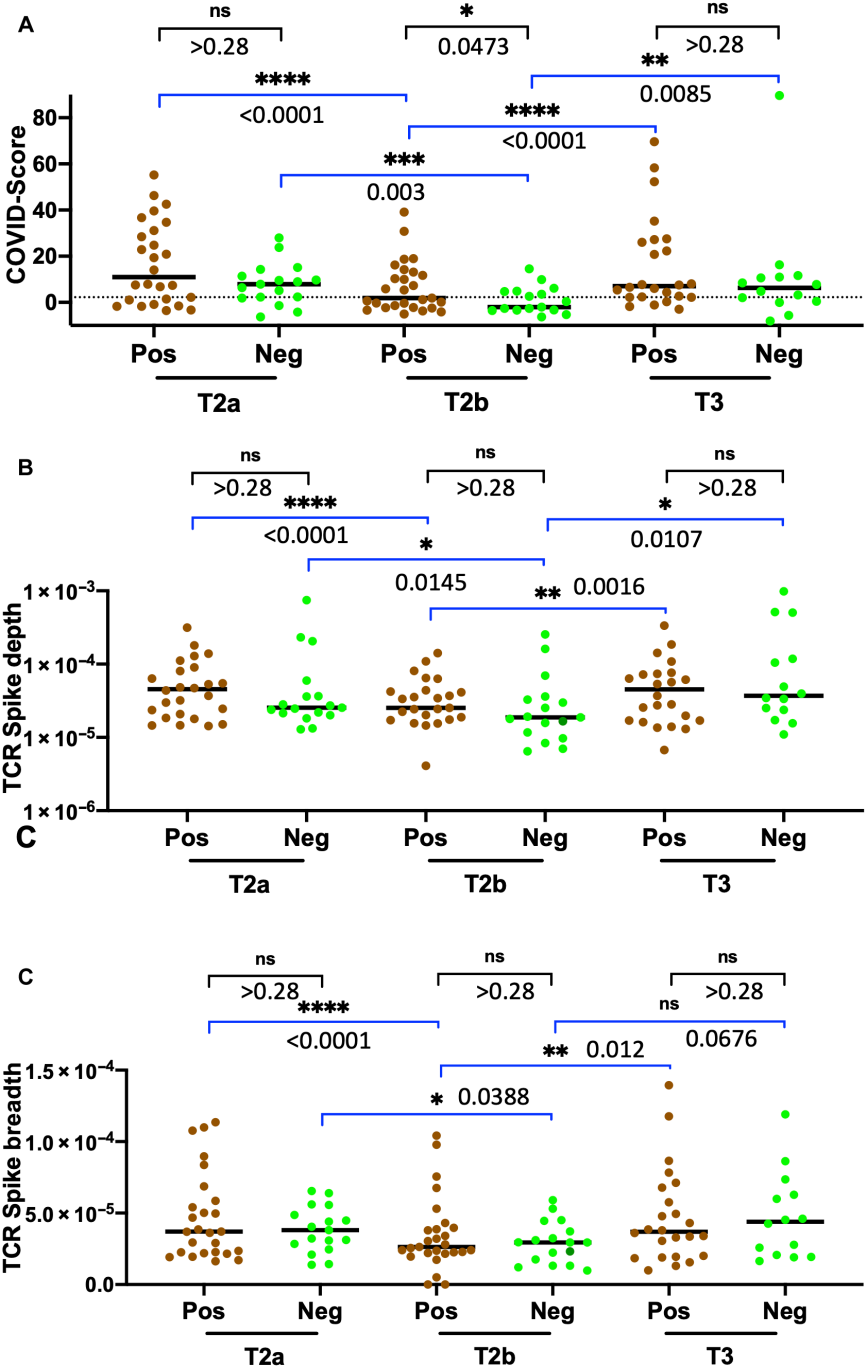
Dynamics of T cell clonal responses before and after the third booster dose. (**A**) Analysis of SARS-CoV-2 TCRβ levels at T2a, T2b, and T3 sampling points for LFA-positive and LFA-negative groups. A dotted line representing the threshold for the TCR sequencing assay (2.23) is shown. Analysis of SARS-CoV-2 Spike TCRβ (**B**) depth and (**C**) breadth at T2a, T2b, and T3 sampling points for LFA-positive and LFA-negative groups. LFA-positive and LFA-negative groups were classified on the basis of the LFA results of the blood samples collected at T2b. The nonparametric Wilcoxon matched pair set summary is presented within the LFA-positive or LFA-negative groups between sampling time points that are shown in blue brackets above the plot. In addition, the Mann-Whitney summary between the LFA-positive and LFA-negative at the respective sampling time points is shown in black brackets above the plot.

Next, to assess differences in T cell response (*38*) among the LFA-positive and LFA-negative groups, we conducted immuno-sequencing of the CDR3 regions of human TCRβ chains. We used a T cell classifier (COVID-Score), previously established for diagnosing SARS-CoV-2 infection (*39*, *40*), to the samples collected at baseline (T0) and after the first dose of the mRNA vaccine (T1) and found that COVID-Score exhibited a high specificity and sensitivity within our HCP cohort (fig. S6). Analysis of T cell response by COVID-Score before (T2a and T2b) and after the third booster dose (T3) showed a trend consistent with previous reports (*41*–*43*) that showed the contraction following two-dose of mRNA vaccine and expansion after the third booster dose (Fig. 6A). Analysis of the T cell receptor (TCR) spike breadth (the percentage of distinct T cell clonal lineages in a repertoire that is specific to SARS-CoV-2 spike protein) and TCR spike depth (which is linked to the relative frequency of SARS-CoV-2 spike protein–specific T cell clones in a repertoire) also followed a similar trajectory in both LFA-positive and LFA-negative groups (Fig. 6, B and C) in line with the kinetics of a healthy T cell response (*44*, *45*). However, unlike the notable difference we observed in the humoral response for the LFA-positive and LFA-negative groups, T cell breadth and depth did not show any statistically notable difference between these two groups before or after the third booster dose.

## DISCUSSION

As new SARS-CoV-2 strains appear with substantial antigenic drift from the ancestral strain (*46*), the optimal strategy may rely on vaccines tailored to the new strains. The 2022 bivalent booster vaccine contained mRNA sequences for the spike protein of both ancestral and Omicron subvariants (BA.4/BA.5), while the newly approved monovalent booster targets XBB.1.5 (*47*). However, uptake of the bivalent booster in the eligible US population was under 20% (*48*) as of May 2023. Even among 65 and older US residents, the bivalent booster uptake has been reported as under 41%, suggesting the need for new strategies to build confidence and promote the use of the SARS-CoV-2 vaccine (*49*). Along these lines, a recent Center for Disease Control and Prevention report recommends using evidence-based strategies to help increase vaccine uptake (*50*).

POC LFAs based on the SARS-CoV-2 antigen were broadly adopted (*51*) during the pandemic for self-diagnosis. They have been proven to play an essential role in saving lives, reducing hospital admissions, and preventing transmission (*52*). There is a growing interest in expanding the use of home-based POC testing (*53*). Since the LFA format is already familiar to people who use LFA antigen (Ag) tests, the acceptance of LFA Ab tests is likely to be high, making it convenient and scalable. In contrast, central lab-based testing requires licensed HCPs to order the tests, and testing can only be done at specific hours and places, which limits its usefulness. POC LFA Ab tests have also been reported as an effective public health tool (*54*) and could serve as a population-level screening platform to preserve herd immunity. As a proof of concept, we used US Food and Drug Administration issued EUA blood- and oral fluid–based lateral flow POC Ab tests to detect inadequate SARS-CoV-2 humoral immunity in a cohort of healthy adults who showed variable neutralizing antibodies following two-dose mRNA vaccination (*4*). The POC test results were rapid (under 20 min); negative results led to definitive agreement on low or absent neutralizing Abs for the Wuhan, Delta, and BA.1 Omicron strains. However, interpretation of POC Ab test results is limited (*22*, *55*). In our study cohort, a positive POC Ab test result was consistent with, but not evidence for, adequate immunity; a negative test was correlated with inadequate immunity and thus can serve as an empiric basis for further vaccination. The utility of this approach is supported by a recent large-scale study which reported that a negative POC LFA Ab test result in those fully vaccinated is associated with substantially increased risk for hospitalization and death (*56*).

Ab-mediated SARS-CoV-2 neutralization mainly occurs through recognizing conformational epitopes on the spike protein (*57*). In macaque challenge studies, neutralizing antibodies protected against SARS-CoV-2 infection in a dose-dependent manner (*58*). A predictive model based on studies of seven different vaccines and convalescent cohorts estimated that neutralization level strongly correlates with immune protection against SARS-CoV-2 in humans (*5*). Similarly, hospitalized patients with COVID-19 who received high tittered (>1:640) neutralizing convalescent plasma were discharged earlier than patients who received convalescent plasma with lower neutralizing Ab titers (*59*). These studies support that higher systemic neutralizing Ab levels predict SARS-CoV-2 protection.

The serial emergence of antigenically distinct SARS-CoV-2 variants has substantially reduced the effectiveness of vaccine-induced neutralizing Abs (*60*). Even so, individuals with high neutralizing Ab titers were more protected against emerging strains than those with weak neutralizing Ab levels (*4*). In our HCP cohort, we observed a notable decrease in neutralizing Ab levels >4 months after the second dose of the mRNA vaccine, although more pronounced in the NV subjects than in the IV subjects. Individuals who developed strong neutralizing Abs against the Wuhan strain shortly after the second dose had modest neutralizing Abs against the Omicron strain six months out (T2b). However, individuals who developed inadequate neutralizing Ab response after the second dose entirely lacked neutralizing Abs against the Omicron strain after ~6 months, suggesting that risk for infection following vaccination is not limited to at-risk groups, including older/immunocompromised recipients (*61*), but is applicable population-wide (*5*, *62*).

After boosting with a Wuhan strain–based mRNA vaccine, binding and neutralizing Abs sharply rose against Wuhan, Delta (B.1.617.2), and Omicron (B.1.1.529) strains for most individuals in our cohort. Notwithstanding that those with positive results outperformed them, the relative gain for subjects who had negative POC LFA Ab tests and inadequate neutralizing Abs after the second dose was notable. This highlights the importance of receiving a booster among those that did not experience a robust neutralizing response after initial two-dose vaccination. Although we did not find evidence for salivary IgA stimulation following the vaccine booster, the salivary IgG strongly tracked the serum IgG levels as recently reported (*63*). It is possible that a sufficient concentration of salivary IgG could potentially block SARS-CoV-2 entry into oral epithelial cells (*64*).

Several studies also reported that Ab avidity increased after booster vaccination against Wuhan and Omicron strains (*61*, *65*, *66*). Ab avidity after the Wuhan-based booster vaccine also improved overall in our study, although for the Wuhan and Delta strains, this change was higher than for Omicron. Our study is also unique in illustrating the improvement in the neutralizing Ab breadth after the third boost (T3) against Bat SARS-like coronavirus RsSHC014, representing a divergent member of the sarbecovirus family. This illustrates immune maturation with repeated exposure to SARS-CoV-2 Ag (*67*) in our cohort and underlines that vaccine-mediated immunity to strains no longer in circulation can nonetheless extend to newer/genetically distinct strains (*68*). However, the Ab avidity toward SHC014 is slightly stronger than against Omicron, suggesting that choice mutations in the RBD region drive escape from neutralizing antibodies.

Cellular immune responses are known to reduce symptomatic and severe disease (*69*) against SARS-CoV-2 infection in the setting of waning and sub-optimal neutralizing Ab titers (*58*); protection against hospitalization and death among individuals experiencing a breakthrough infection with a SARS-CoV-2 variant is high (*70*), suggesting that T cells play a substantial role against severe disease in the context of infection by SARS-CoV-2 variants (*71*). In our cohort of healthy adults, T cell clonal levels of TCR breadth and depth followed a typical trend reflective of healthy T cell response (*38*), and there were no notable differences among LFA-positive and LFA-negative individuals at any sampling point. A comparative study of a healthy adult cohort that analyzed the dynamics of T cell stimulation to produce cytokines showed that T cell immunity is established and maintained after complete vaccination, as the T cell effector (*72*) function remained stable before and after the booster vaccination (*73*). Moreover, TCR reactivity occurs through presentation of a broad variety of viral peptides displayed in combination with human leukocyte antigens on the surface of infected cells with the results that T cell responses are less sensitive to mutations in the spike protein (*74*, *75*). These observations support the idea that booster vaccination may be more crucial for improving neutralizing Ab levels than T cell responses (*73*).

Overall, our findings show that those with adequate immunity, as well as those with inadequate immunity, gained neutralizing antibodies to distantly related SARS-CoV-2 variants and related SARS-like viruses through booster vaccination. We found that repeated vaccine doses help the low-responding vaccinees gain depth and breadth in plasma Abs to increase protection against highly evolving SARS-CoV-2 strains. As has been shown in a recent population-based study (*56*), negative POC LFA Ab tests indicate increased risk for severe outcomes. While a positive POC LFA Ab test result does not indicate adequate immunity, a negative POC LFA Ab test can inform people about inadequate immunity leading people to obtain booster vaccinations which can, in turn, be life-saving and/or keep them from developing severe disease requiring hospitalization and potential long-lasting health effects.

A limitation of our longitudinal cohort study is that our participants only include primarily young, healthy adults; also, our cohort does not contain children, chronically ill, or immunocompromised subjects. Our feasibility study has yet to incorporate subjects after the bivalent booster vaccine, and the commercial POC tests used were based on the spike antigen of the Wuhan strain. Nevertheless, our study evaluated the depth and breadth of cellular and Ab responses in systemic and oral compartments. We showed that vaccine boosting with the third dose enhances Ab titers, neutralization activity, and ACE2 blockage and heightens Ab avidity across heterogeneous subjects, whose response varied after the second dose. We also presented a proof of concept showing how LFA POC tests with appropriately updated RBD antigens for circulating strains can inform those with inadequate immunity about the need for booster vaccination. In addition, it may be challenging to interpret results from current LFA Ab tests as they are primarily designed to detect exposure to SARS-CoV-2. Redesigning the tests to specifically detect negative Ab results with clear instructions for interpreting those results will prevent any positive results that could mislead people into believing they are completely protected against COVID-19.

## MATERIALS AND METHODS

### Clinical study and specimen collection

We enrolled a total of 237 HCPs working at the emergency department (ED) of George Washington University Hospital (GWUH). Emails for recruitment were sent to all GWUH ED HCP personnel through an ED staff listserv. In addition, to reach those not on the listserv, notifications were sent out through GWUH’s ED nurse/technician scheduling system, and fliers were placed in break rooms with quick response codes connecting to patient sign-up forms. Participation days and times overlapped nurse and technician shift changes to encourage ongoing and off-going staff to participate. The clinical roles of study participants included physicians, advanced practice providers, nurses, and ED technicians. The study was approved by the George Washington University Institutional Review Board no. NCR202406. All ED HCP personnel participating in this study provided written informed consent. All personnel who consented to participate were included in the study. Samples were collected in May/June 2020 (baseline, T0), January 2021 (after the first dose, T1), March 2021 (after the second dose, T2a), July/August 2021 (after the second dose/before booster, T2b), and November/December 2021 (after the booster, T3). Not all participants provided specimens at every sampling point. Also, laboratory analyses were not performed if the vaccination dates were unknown or the booster was received before T2b sampling point. Venous blood samples were collected into a serum separation tube, refrigerated overnight to allow for serum separation, and stored at −80°C until laboratory analysis. Saliva samples were collected into a specialized device (Oracol S14 saliva collection device, Malvern Medical Developments, UK) according to the manufacturer’s instructions and stored at −80°C until laboratory analysis. Both polymerase chain reaction (PCR) and nucleocapsid Ab test were used to determine the past SARS-CoV-2 infection history of the subjects.

### Live-virus neutralization assay

Neutralization assays were conducted using full-length live reporter virus constructs of Wuhan SARS-CoV-2 D614G [sequence accession (Ac). no. MT020880], Delta SARS-CoV-2 B.1.617.2 Ac. no. OV116969.1), Omicron SARS-CoV-2 B.1.1.529 (EPI_ISL_6647961), and SHC014-Bat-CoV (Ac. no. KC881005.1) with the nano-luciferase (nLuc) reporter gene replacing open reading frame 7a (ORF 7a) in SARS-CoV-2 variants and ORF 8 in SHC014 as previously published reports (*18*, *23*, *76*). Assays were conducted in a modified manner from previously reported study (*18*). Heat-inactivated serum samples were initially diluted 1:20 and then serially diluted fivefold down a 96-well plate (Corning 3799) in virus growth medium [1× minimum essential medium (Gibco 11095080), 5% fetal bovine serum (FBS) (Hyclone SH30070.03HI), and 1% penicillin-streptomycin (Gibco 10378016)] before transfer to the Biosafety Level 3 (BSL3) laboratory for assay completion. In the BSL3 laboratory, nLuc viruses were individually diluted in the virus growth medium, added in equal volume to serum dilution plates, and incubated for 1 hour at 37°C, 5% CO_2_. The virus and serum dilutions were then added to duplicate columns of a 96-well black plate (Corning 3916) seeded 1 day prior with 2 × 10^4^ Vero C1008 cells per well for a final virus dilution of 800 plaque-forming units per well and incubated at 37°C, 5% CO_2_. After 20 to 24 hours, the virus was quantified with the Promega Nano-Glo Luciferase Assay system (N1130) with a Promega GloMax Explorer (GM3500). 50% inhibitory concentration was defined as the serum dilution at which the observed relative light units were reduced by 50% compared to virus^+^ cell and virus-only control wells as determined by an Excel macro and analyzed using GraphPad Prism 9.3.1.

### HCoV focus reduction neutralization test

NL63, OC43, and 229E viruses were grown and tittered on LLC-MK2 cells. To measure Ab that mediate neutralization, fourfold serial dilutions of human serum were mixed with ∼70 focus-forming units of virus, incubated at 37°C for 1 hour, and added to LLC MK2 monolayers in 96-well plates for 1 hour at 37°C to allow virus adsorption. Cells were overlaid with 2% methylcellulose mixed with Dulbecco’s modified Eagle’s medium containing 5% FBS and incubated for 24 hours at 37°C. Medium was removed, and the monolayers were fixed with 5% paraformaldehyde in phosphate-buffered saline (PBS) for 15 min at room temperature, rinsed, and permeabilized in Perm/Wash buffer (PBS and 0.05% Triton X-100). Infected cell foci were stained by incubating cells with polyclonal anti-NL63 (Sino Biological, 40641-T62), anti-OC43 (Sino Biological, 40643-T62), and anti-229E (MAB10938) for 1 hour at 37°C and then washed three times with Perm Wash. Foci were detected after the cells were incubated with a 1:5000 dilution of horseradish peroxidase–conjugated goat anti-rabbit IgG for NL63 and OC43 and 1:5000 dilution of horseradish peroxidase–conjugated goat anti-mouse IgG (Sigma-Aldrich) for 1 hour. After three washes with Perm/Wash buffer, staining was visualized by addition of TrueBlue detection reagent [SeraCare (Kirkegaard & Perry Laboratories)]. Infected foci were then enumerated by Cellular Technology Limited ELISPOT. Focus reduction neutralization test curves were generated by log transformation of the *x* axis followed by nonlinear curve fit regression analysis using GraphPad Prism 8.

### Multiplex SARS-CoV-2 IgG and sIgA assays for oral fluid

Oral fluid IgG and sIgA to SARS-CoV-2 were measured with a multiplex immunoassay as described previously (*77*). Briefly, after thawing and centrifuging oral fluid for 5 min at 10,000*g*, the 10-μl supernatant was added to each microtiter well that contained 1000 coupled beads per bead set in 40-μl assay buffer [Phosphate-buffered saline with 0.05% Tween-20, 0.1% bovine serum albumin (BSA) and sodium azide]. Each multiplex IgG plate contained a blank well with assay buffer instead of sample for background correction, high and low SARS-CoV-2 IgG–positive controls, and a SARS-CoV-2 IgG–negative control in addition to an in-house SARS-CoV-2 IgG standard curve. Phycoerythrin-labeled antihuman IgG diluted 1:100 in assay buffer was used to detect the IgG signal in saliva (Jackson ImmunoResearch, 109-115-098). A monoclonal antihuman secretory component Ab, followed by a phycoerythrin-labeled anti-mouse Ab, was used to detect anti–SARS-CoV-2 sIgA. The plate was read on a Luminex MAGPIX instrument.

### T cell receptor variable β chain sequencing

Immunosequencing of the CDR3 regions of human TCRβ chains was performed using the immunoSEQ Assay (Adaptive Biotechnologies, Seattle, WA). Extracted genomic DNA was amplified in a bias-controlled multiplex PCR, followed by high-throughput sequencing. Sequences were collapsed and filtered to identify and quantitate the absolute abundance of each unique TCRβ CDR3 region for further analysis as previously described (*78*–*80*). The fraction of T cells was calculated by normalizing TCRβ template counts to the total amount of DNA usable for TCR sequencing. The amount of functional DNA was determined by PCR amplification and sequencing of several reference genes that are expected to be present in all nucleated cells.

### Mapping of SARS-CoV-2 TCRβ sequences

TCR sequences from repertoires were mapped against a set of TCR sequences known to react to SARS-CoV-2. These sequences were first identified by Multiplex Identification of T cell Receptor Antigen (MIRA) Specificity (*81*). TCRs that react were further screened for enrichment in COVID-19–positive repertoires collected as part of immuneCODE (*39*) compared to COVID-19–negative repertoires to remove TCRs that may be highly public or cross-reactive to common antigens. The individual response could be quantified by the number and frequency of SARS-CoV-2 TCRs seen postvaccination. TCRs were further analyzed at the level-specific ORF or position within ORF based on the MIRA antigens.

### Recombinant protein antigens

The expression and purification of SARS-CoV-2 full-length spike ectodomain (16 to 1208 amino acids, accession: P0DTC2.1), HaloTagged RBD (331 to 528 amino acids), and nucleocapsid antigens were as previously described (*4*, *25*, *82*). The HaloTagged SARS-CoV-2 RBD antigens for bat SARS-like coronavirus RsSHC014, Delta SARS-CoV-2 B.1.617.2, and Omicron SARS-CoV-2 B.1.1.529 were designed and expressed in mammalian Expi293 cells as described for RBD antigens (*4*, *25*, *82*). RBD antigens were site-specifically biotinylated using HaloTag PEG-biotin ligand (Promega G8281), following the manufacturer’s protocol. The purified full-length ectodomain of the HCoVs spike proteins (HCoV-NL63, 40604-V08B; HCoV-OC43, 40607-V08B; HCoV-229E, 40605-V08B; and HCoV-HKU1, 40606-V08B) was purchased from Sino Biological.

### Spike ELISA

Full-length spike ELISA was performed as described before (*82*). Briefly, full-length spike protein at 2 μg/ml in tris-buffered saline (TBS) (pH 7.4) was coated in a high-binding microtiter plate (Greiner Bio-One 655061) and for 1 hour at 37°C and then blocked with blocking solution [3% milk in 0.05% TBST (TBS and 0.05% Tween 20)] for 1 hour at 37°C. Serum samples were serially diluted (1:33 to 1:8100) in blocking solution, then added to the plate, and then incubated for 1 hour at 37°C. The plate was washed using a BioTek 405 LS microplate washer, and horseradish peroxidase–conjugated secondary goat antihuman secondary Ab IgG at 1:40,000 in 3% milk TBST blocking solution was added for 1 hour at 37°C. After washing the plate, 50 μl of 3,3′,5,5′-tetramethylbenzidine (TMB) liquid substrate (Sigma-Aldrich, T0440) was added, and absorbance was measured at 450 nm after stopping the reaction with 50 μl of 1 N HCl. The area under the curve was calculated using GraphPad Prism.

### RBD ELISA

RBD ELISA was performed as described before (*4*). High-binding microtiter wells were coated with streptavidin (Invitrogen, 434302) in TBS (4 μg/ml) (pH 7.4), incubated for 1 hour at 37°C, then blocked with Non-Animal Protein-BLOCKER (G-Biosciences, 786190 T) in TBS, and incubated for 1 hour at 37°C. Serially diluted serum samples (1:33 to 1:8100) were prepared in a 3% BSA solution with TBST containing 3% BSA and biotinylated RBD antigen (1 μg/ml) and then incubated in a nonbinding 96-well plate for 1 hour at 37°C. Following incubation, the diluted serum was added to the microtiter assay plate where it was incubated for 15 min at 37°C. After washing, horseradish peroxidase–conjugated secondary goat antihuman secondary IgG Ab at 1:40,000 dilution in 3% milk TBST was added, and the plate was incubated for 40 min at 37°C. After washing, TMB liquid substrate (Sigma-Aldrich) was used to develop the color. Absorbance was measured at 450 nm after stopping the reaction with 50 μl of 1 N HCl. A threefold serially diluted anti–SARS-CoV-2 Spike RBD monoclonal Ab (mAb; Abeomics, ABMX-002) was used in every assay plate. A standard curve obtained from the spike RBD mAb was used to define the concentration of Abs in the clinical samples [one binding Ab unity = mAb (1 ng/ml)].

### Nucleocapsid ELISA

The SARS-CoV-2 nucleocapsid IgG ELISA was performed as previously described (*4*). High-binding microtiter wells (Greiner Bio-One, 655061) were coated with anti-maltose–binding protein (MBP) mAb (3 μg/ml; E8032, New England Biolabs), incubated for 1 hour at 37°C, and then blocked with blocking solution (3% nonfat powdered milk in TBST). After washing the plate with TBS containing 0.2% Tween 20, MBP-fused full-length nucleocapsid (2 μg/ml) or MBP protein in blocking solution was added to respective wells and incubated for 1 hour at 37°C. After washing the plate, heat-inactivated serum at a 1:40 dilution was added and incubated for 1 hour at 37°C. The plate was washed, and alkaline phosphatase–conjugated secondary goat antihuman anti-IgG (Sigma-Aldrich, A9544) was added to the wells at 1:2500 dilution. Absorbance was measured at 405 nm after adding the SIGMAFAST p-Nitrophenyl phosphate substrate (Sigma-Aldrich, N2770). Appropriate control sera were included in the study. The nucleocapsid binding signal for each serum was calculated by subtracting the absorbance of the background signal obtained from MBP wells.

### Avidity index of IgG Abs

High-binding microtiter wells were coated with streptavidin at 4 μg/ml in TBS (pH 7.4) for 1 hour at 37°C and then blocked with Non-Animal Protein-BLOCKER (G-Biosciences) in TBS and incubated for 1 hour at 37°C. The plate was washed three times with wash buffer (TBS containing 0.2% Tween 20), and a TBST buffer containing 3% BSA and biotinylated RBD at 1 μg/ml was added and incubated for 30 min at 37°C. After the plate was washed, heat-inactivated serum samples at 1:900 in TBST containing 3% BSA were added and incubated for 1 hour at 37°C. After washing the plate with wash buffer, wells were incubated with and without 4 M urea containing TBST + 3% BSA for 25 min at 37°C. Urea solution was dumped and washed three times, and horseradish peroxidase–conjugated secondary goat antihuman secondary IgG Ab at 1:40,000 dilution in 3% milk was added. The plate was incubated for 40 min at 37°C. TMB liquid substrate (Sigma-Aldrich) was used for signal development, and absorbance was measured at 450 nm after stopping the reaction with 50 μl of 1 N HCl. The avidity index was calculated as the absorbance in the presence of urea/absorbance in the absence of urea.

### Multiplex surrogate neutralization assay

A multiplexed Meso Scale Discovery (MSD) immunoassay (MSD, Rockville, MD) was used to measure the ACE2 blocking Abs to SARS-CoV-2 Wuhan and other variants, including Alpha (B.1.1.7), Beta (B.1.351), Delta (B.1.617.2), IHU (B.1.640.2), and Omicron subvariants (BA1, BA2, and BA3), using the MSD V-PLEX SARS-CoV-2 Panel 25 as previously described (*4*). Briefly, plates were blocked with MSD Blocker A for 30 min and washed thrice. Then, the reference standard, controls, and heat-inactivated samples diluted 1:100 in the diluent buffer were added. Plates were incubated at room temperature for 1 hour with shaking at 700 rpm. MSD SULFO-tag conjugated ACE2 (0.25 μg/ml) was added and incubated for 1 hour at room temperature with shaking. Plates were washed and read with a MESO QuickPlex SQ 120 instrument. ACE2 blocking activity was calculated using the equation: [(1 − average sample ECL signal/average ECL signal of the blank well) × 100].

### LFA assay with blood samples

The Cellex qSARS-CoV-2 IgG/IgM Rapid test was performed following the manufacturer’s instructions. Briefly, 10 μl of blood sample was dispensed into the center of the sample disposal, followed by two drops of sample diluent provided by the kit were added to the sample disposal. The cassette was placed horizontally on a clean, flat surface, and the reaction was left to develop at room temperature for 20 min. The color changes in the test and control lines at 20 min were read by the naked eye, and a photograph was taken for record. A sample was considered positive if both test IgG or IgG + IgM and control line (C) were visible, negative if only “C” line was visible and there was no test line. The test was invalid if the C line failed to develop. The Cellex qSARS-CoV-2 IgG/IgM Rapid test was reported to have a positive predictive agreement (PPA) of 93.8% and a negative predictive agreement (NPA) of 96.0% using plasma samples from reverse transcription PCR–positive individuals.

### LFA assay with oral fluid samples

The CovAb SARS-CoV-2 Ab Rapid Test was performed with oral fluid samples described by manufacturer instructions on a clean, flat surface. Briefly, five drops of saliva sample were diluted with the buffer provided by the manufacturer’s kit at a 1:1 ratio and then transferred to the sample well, allowing the reaction to run for 15 min. The visible test and control lines were read at the end of 15 min, and a photograph was taken for record. A sample was considered positive if both test and control lines were visible and negative if only a control line was visible and the test line was absent. The test was invalid if the C line failed to develop. The CoV Ab test was reported to have a PPA of 97.1% and an NPA of 97.4% when used for POC testing with oral fluid samples collected more than 15 days after the onset of symptoms.
