# Supplementary material for: COVID-19 point-of-care tests can identify low-antibody individuals: In-depth immunoanalysis of boosting benefits in a healthy cohort
Source: Sci Adv. 2024 Jun 12;10(24):eadi1379. doi: 10.1126/sciadv.adi1379 (PMC11168476; doi:10.1126/sciadv.adi1379)
Supplement: Supplementary file 1 — Figs. S1 to S5 [file sciadv.adi1379_sm.pdf]

Science Advances
AAAS

Supplementary Materials for

COVID-19 point-of-care tests can identify low-antibody individuals: In-depth immunoanalysis of boosting benefits in a healthy cohort

Michael Mallory *et al.*

Corresponding author: John E. Lafleur, jlafleur@mfa.gwu.edu; Ralph S. Baric, rbaric@unc.edu; Lakshmanane Premkumar, prem@med.unc.edu

Sci. Adv. **10**, eadi1379 (2024)
DOI: 10.1126/sciadv.adi1379

This PDF file includes:

Figs. S1 to S5

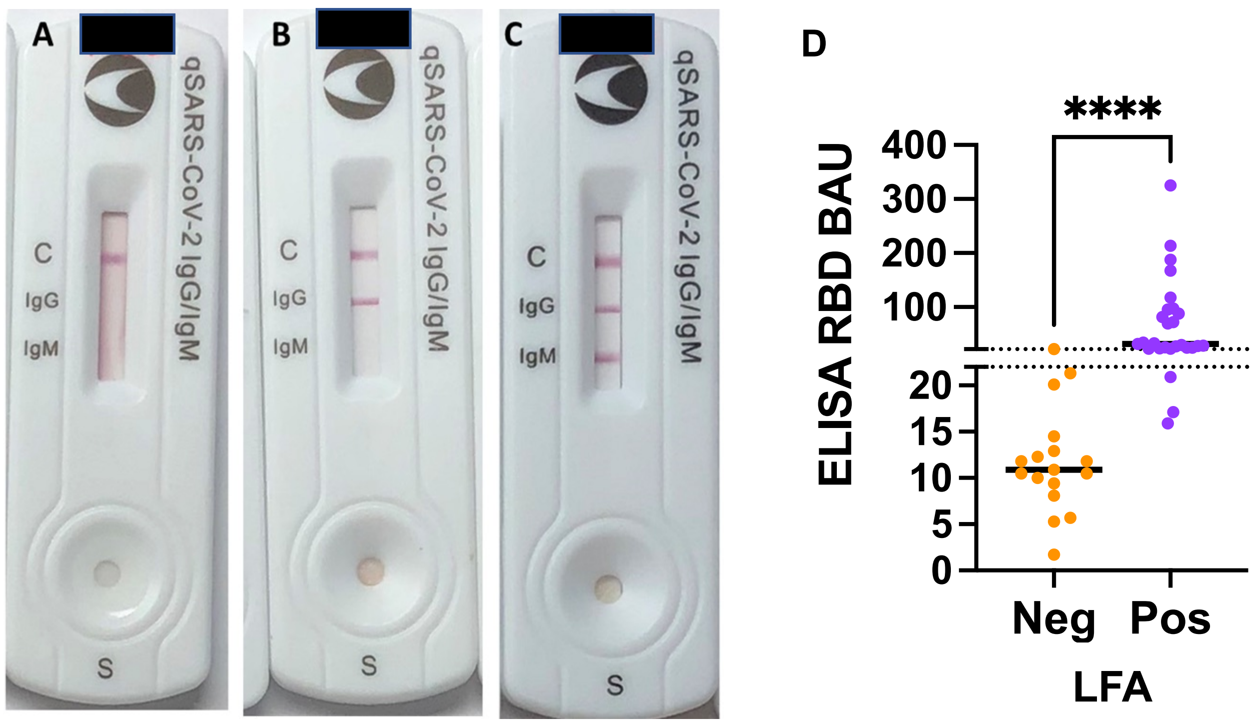

Fig. S1. CELLEX LFA test results with serum samples for (A) valid negative (B) valid IgG positive and (C) valid IgG+IgM positive readings. (D) ELISA RBD binding agreements with the LFA blood test.

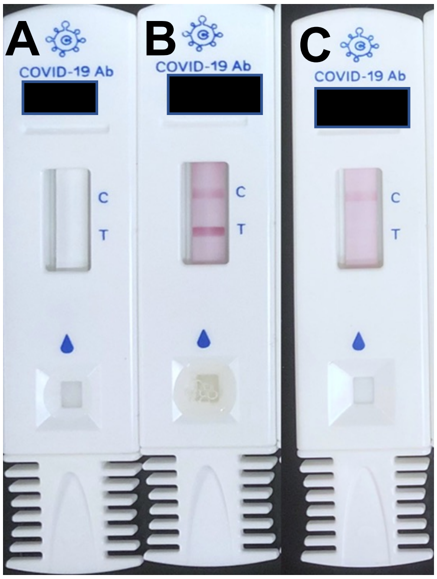

Fig. S2. COVAB LFA test results with salivary samples for (A) invalid (B) valid positive and (C) valid negative readings.

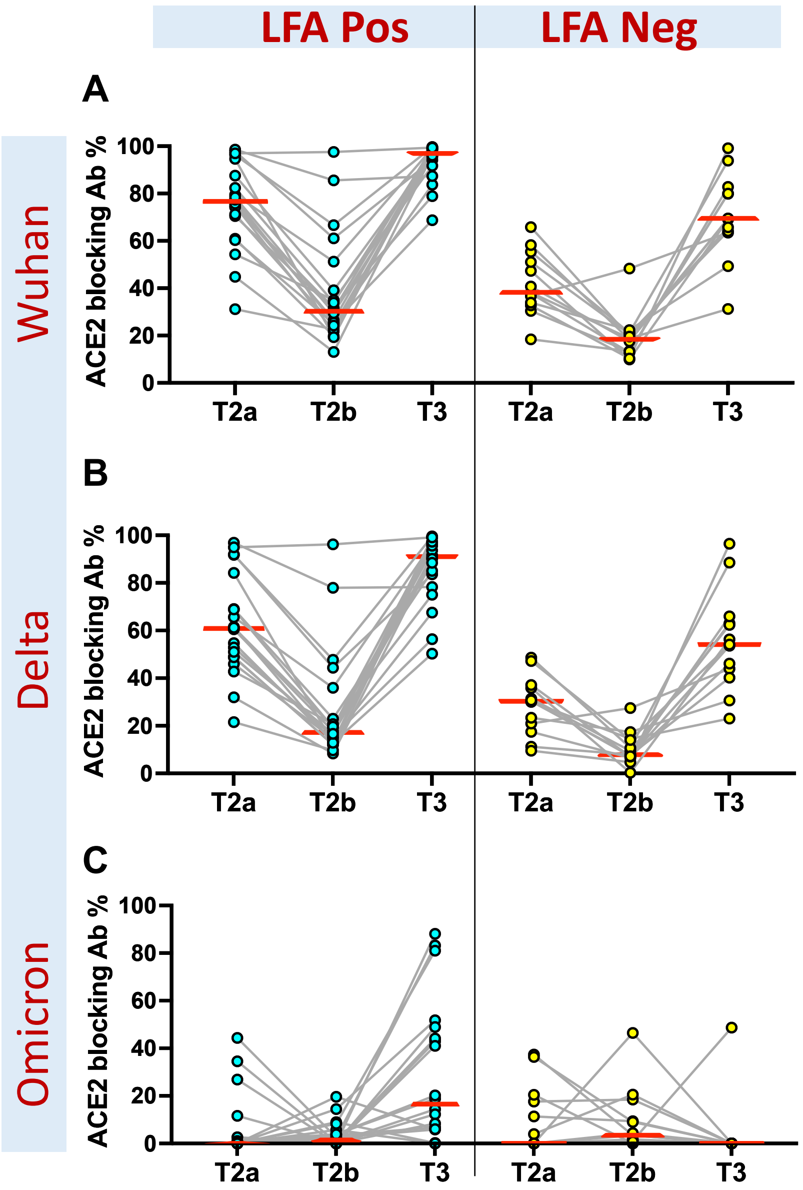

Fig. S3. Dynamics of ACE2 blocking Ab responses before and after the third booster dose. Analysis of (A-C) ACE2 blocking Abs to the Wuhan, Delta, and Omicron variants at T2a (4-9 weeks after dose-2), T2b (19-36 weeks after dose-2) and T3 (3-18 weeks after dose-3). LFA positive and negative groups were classified based on the LFA results with the T2b blood sample.

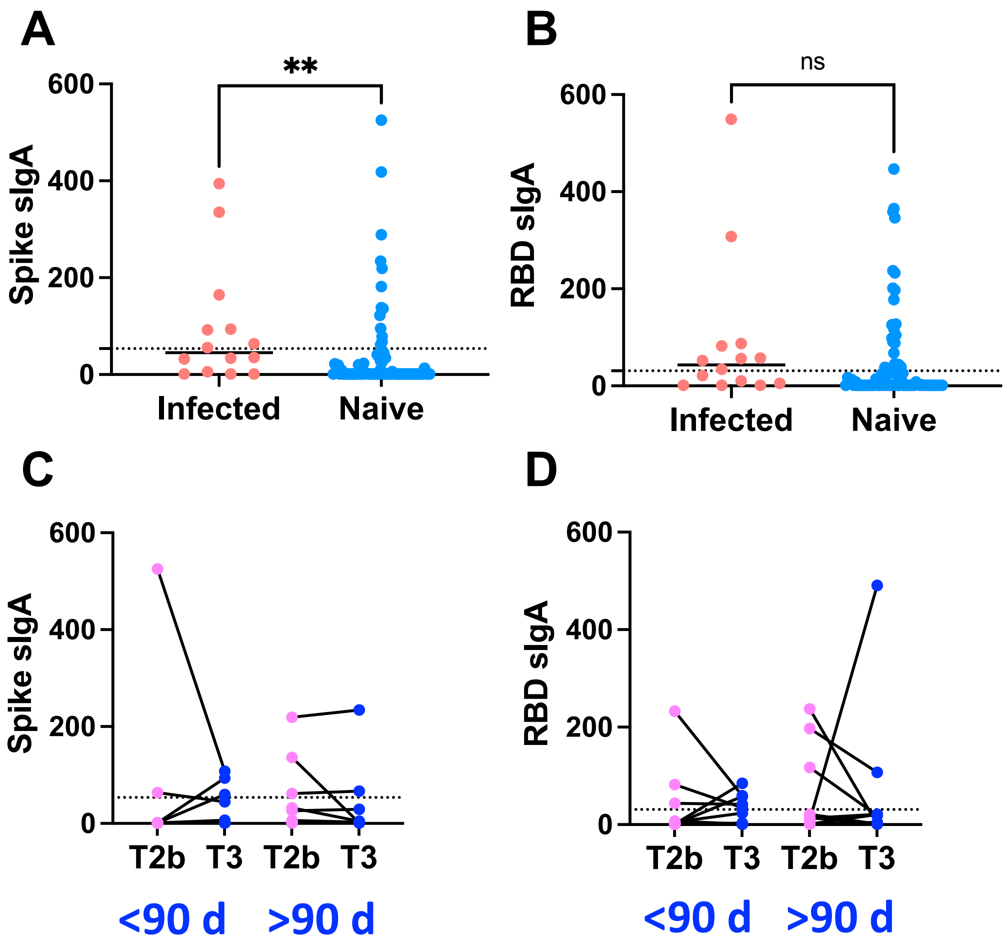

Fig. S4. Absence of secretory IgA (sIgA) Abs following the mRNA vaccine booster. Comparison of sIgA levels in infected-vaccinated and naive-vaccinated subjects against (A) Spike and (B) RBD antigens. sIgA levels were assessed by a multiplex Luminex assay in oral fluid samples (n=14, infected vaccinated and n=77, naive-vaccinated) collected at pre- and post-booster. A Mann-Whitney non-parametric test to determine if sIgA levels differ between the groups showed that the infected vaccinated group had a modestly higher anti-spike sIgA level than the naïve boosted group (p=0.0021). However, the difference between these groups was insignificant in anti-RBD sIgA levels. Assessment of sIgA levels in paired pre- and post-booster samples against (C) Spike and (D) RBD antigens. The sIgA levels to spike and RBD in post-booster samples, collected <90 days from 15 subjects or collected >90 days post-booster from 9 subjects, were compared to their respective pre-booster levels. As the Wilcoxon-non-parametric paired test indicated, the sIgA levels in pre- and post-booster paired samples were comparable, suggesting a lack of evidence for sIgA development after the 3rd booster shot. The plot's dotted line represents the sIgA assay thresholds for Spike sIgA (53.6 MFI, BSA background subtracted) and RBD (31 MFI, BSA background subtracted), determined based on the reference panel.

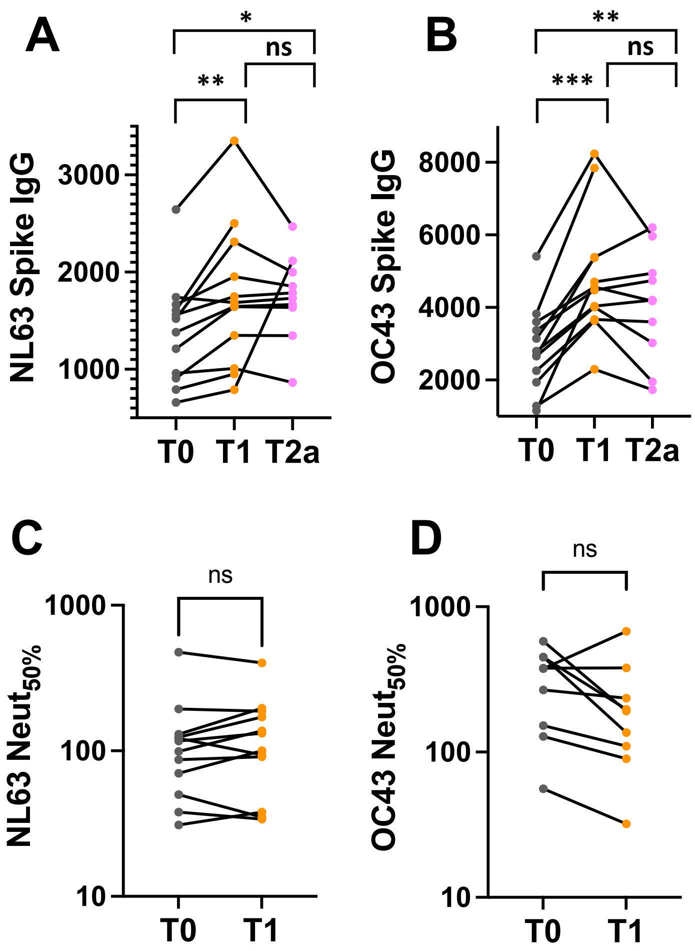

Fig. S5. HCoV CR reactive Abs induced following mRNA vaccine lacks cross-neutralizing activity. Analysis of spike IgG Abs to (A) NL63 and (B) OC43 at pre-vaccine and after doses 1 and 2. Binding IgG antibodies were measured by ELISA using serially diluted sera from 12 subjects collected at pre-vaccine, after dose 1 (2-4 weeks), and after dose 2 (5-9 weeks) and expressed as area under the curve (AUC). Analysis of neutralizing Abs to (C) NL63 and (D) OC43 at pre-vaccine and after doses 1 and 2. Wilcoxon-non-parametric paired test was performed to determine if antibody response to NL63 and OC43 between baseline and after doses differ. The test result showed that even though the spike binding antibody to seasonal CoVs is elevated between baseline and after vaccination, they do not effectively contribute to cross-neutralization. The p-value summary from the Wilcoxon test is shown above the scatterplot. p < ∗∗∗0.0005; ∗∗0.0020; ∗0.0332; ns, >0.075.
